# Whole-genome sequence of a varicella-zoster strain from an immunocompetent pediatric patient with severe herpes zoster

**DOI:** 10.1128/mra.01123-25

**Published:** 2026-05-18

**Authors:** Alejandro Ortigas-Vasquez, Braylen V. Washington, Mackenzie Shipley, David C. Bloom, Charles Grose, Moriah L. Szpara

**Affiliations:** 1 Department of Biology, Pennsylvania State University 8082, University Park, Pennsylvania, USA; 2 Center for Infectious Disease Dynamics, Huck Institutes of the Life Sciences, Pennsylvania State University 8082, University Park, Pennsylvania, USA; 3 National Synthesis Center for Emergence in the Molecular and Cellular Sciences, Pennsylvania State University 8082, University Park, Pennsylvania, USA; 4 Department of Microbiology and Cell Science, University of Florida College of Agricultural and Life Sciences 124504, Gainesville, Florida, USA; 5 Department of Biochemistry and Molecular Biology, Pennsylvania State University 8082, University Park, Pennsylvania, USA; 6 Department of Molecular Genetics and Microbiology, University of Florida College of Medicine 12233, Gainesville, Florida, USA; 7 Department of Pediatrics, Division of Infectious Diseases/Virology, University of Iowa 4083, Iowa City, Iowa, USA; Katholieke Universiteit Leuven, Leuven, Belgium

**Keywords:** herpesvirus, genomics, varicella vaccines, varicella-zoster virus, vaccines

## Abstract

We report the complete genome sequence of CGI, a strain of varicella-zoster virus (VZV) isolated from a 3-year-old immunocompetent patient with severe herpes zoster. The isolated strain exhibits an ORF62 genotype characteristic of vOka, in addition to a wild-type ORF0 gene and a novel methionine to valine substitution in ORF58.

## ANNOUNCEMENT

Varicella-zoster virus (VZV) or human alphaherpesvirus 3 (*Orthoherpesviridae*, *Varicellovirus humanalpha3*) causes chickenpox (varicella) and shingles (herpes zoster) (1). The live-attenuated strain vOka is widely used as a vaccine to immunize against VZV (2).

Via Dr. Charles Grose (University of Iowa; CGI), the VZV strain CGI was first documented as “Case 1” in reference 3 and summarized as “Case 17” in reference 4. This sample came from a child previously immunized with vOka (19 months earlier), who presented with severe zoster on his leg (4). A vesicular swab was collected, pelleted, Proteinase K treated (Qiagen), and DNA extracted (DNeasy Kit; Qiagen) (3). After obtaining University of Iowa Institutional Review Board approval (IRB# 201903815), DNA was shipped to the Pennsylvania State University and quantified via Qubit dsDNA High-Sensitivity Assay (Invitrogen). After acoustic shearing (to ~500 bp; Covaris Ultrasonicator M220), library preparation was performed (KAPA LTP Library Kit; Roche) with 14 PCR cycles (5). Oligo-enrichment for viral DNA used published SeqCap baits (5) (Roche) following the SeqCap EZ Library SR User’s Guide v4.2. After PCR amplification (14 cycles), libraries were quality-checked by Qubit and qPCR and sequenced using v3 paired-end chemistry (300 × 300 bp) on an Illumina MiSeq following manufacturer’s instructions.

VZV-specific reads were identified using Kraken2 v2.1.6 (6), followed by quality control and preprocessing via our published viral genome assembly (VirGA) workflow (Step 1), to remove adapters and trim low-quality bases (7). Default settings were used for all software unless otherwise specified. The resulting 7,723,996 forward + reverse reads were used for *de novo* assembly via both metaSPAdes v3.14.0 and SSAKE v4.0 with Celera v8.2 (VirGA Step 2) (8–10). Assembly statistics were assessed using QUAST v5.3.0 (11). metaSPAdes yielded six contigs (130,188 bp total), while SSAKE/Celera generated 26 contigs (161,853 bp total). Each set of contigs was input to VirGA steps 3 and 4 for genome linearization, annotation, and quality assessment (7). The resulting pair of draft genomes was aligned using MAFFT v7.505 in Geneious Prime v2025.1.3 (12, 13). The final genome sequence was constructed by taking repetitive regions from the SSAKE/Celera assembly and curating the corresponding positions in the metaSPAdes assembly. A BAM file for the final genome was generated by mapping quality-controlled reads using Bowtie 2 v2.5.4 (14).

**Fig 1:**
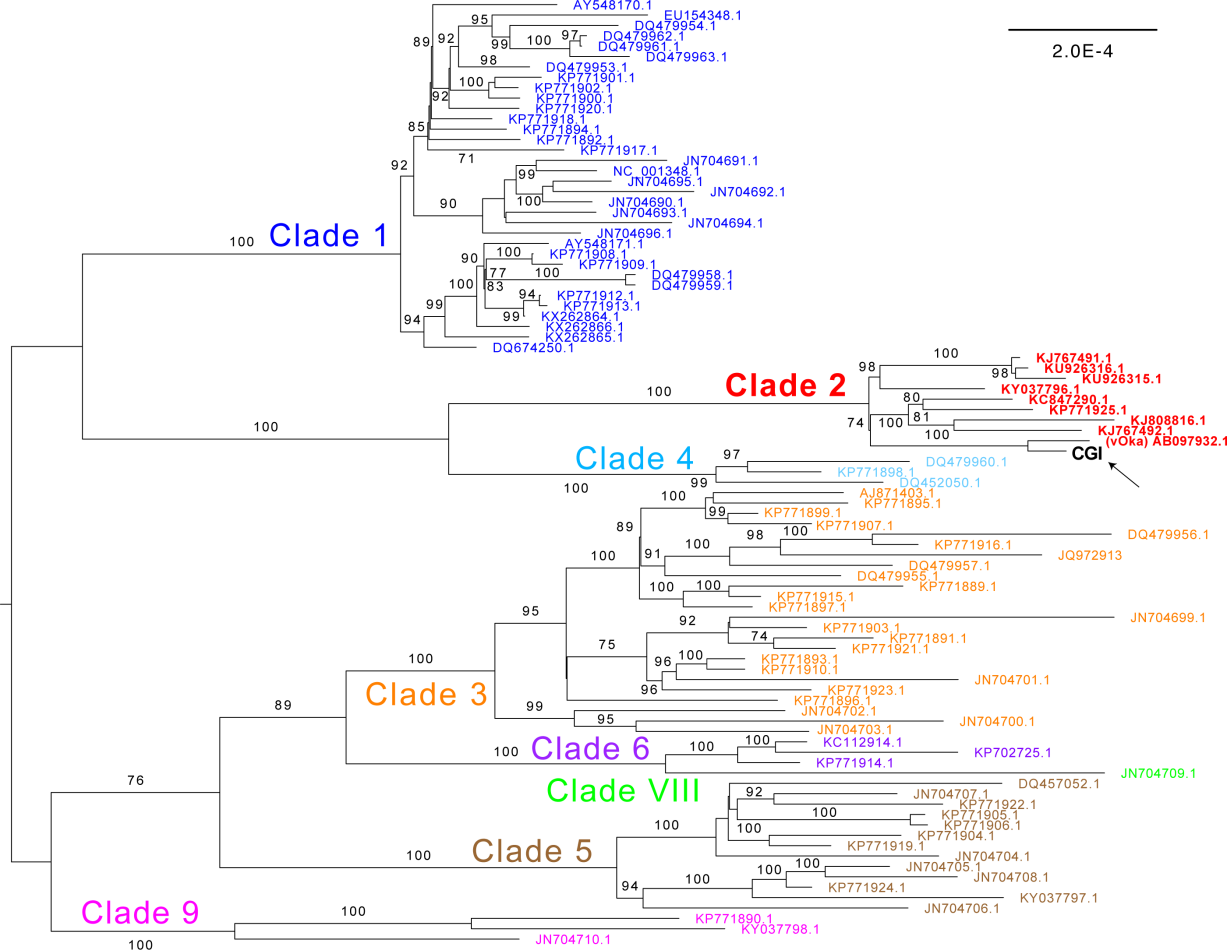
CGI falls in Clade 2 and is closest genetically to vOka**.** A trimmed (terminal repeats excluded) multiple-sequence nucleotide-based alignment of the newly sequenced CGI strain with 90 previously published VZV genomes (15) was generated using MAFFT. A maximum-likelihood tree was constructed using IQTREE v3.0.1 (TVM + F + I + R4 model, 1,000 bootstrap replicates) and visualized using FigTree v1.4.4 (16). The Bayesian Information Criterion (BIC) in IQ-TREE was used to determine the most suitable substitution model. Bootstrap values > 70 are shown. Each Clade (I–VIII) is highlighted with a different color. CGI (black, indicated by an arrow) is located in Clade 2 (red, bold) and appears closest genetically to the VZV vaccine strain vOka. The nucleotide sequences of CGI and vOka (GenBank: AB097932.1) were then separately aligned using MAFFT. The resulting alignment was visualized using Geneious Prime to enable pairwise comparisons, revealing a total of 354 base-pair differences (141 SNPs) for a total of 99.6% shared identity. Total differences and percent identities were calculated using the Geneious Prime nucleotide statistics tool.

The final genome of the VZV strain “CGI” was 125,047 bp in length, with 46.1% GC content (GenBank: PX436161) and an average coverage of 7,630×. To assess genome completeness and verify genome boundaries, VZV CGI was aligned to reference strain Dumas (GenBank: NC_001348.1) using MAFFT and the alignment visualized in Geneious Prime. Phylogenetic analysis revealed that CGI belonged to Clade 2, with VZV vaccine strain vOka as its nearest neighbor (Fig. 1).

CGI exhibits the characteristic nucleotide pattern of vOka in ORF62 (17, 18). CGI also exhibits a wild-type codon for residue 130 of ORF0, which encodes a stop codon found in wild-type VZV strains (19). This ORF0 stop codon is replaced by an arginine in VZV vaccine and attenuated strains, such as vOka and Ellen (20). In addition, CGI shows a unique thymine to cytosine polymorphism in nucleotide 229 of ORF58 (i.e., methionine to valine at residue 77). A BLAST search confirmed that this polymorphism has not been previously observed in any other published VZV sequences.

## Data Availability

Raw sequencing data are available in the Sequence Read Archive (BioProject: PRJNA1335295), and the complete nucleotide sequence is under GenBank accession number PX436161.
